# Preface

**DOI:** 10.2188/jea.15.S87

**Published:** 2005-08-18

**Authors:** Takesumi Yoshimura, Yutaka Inaba, Yoshinori Ito, Shuji Hashimoto, Akiko Tamakoshi, Yoshiyuki Watanabe

**Affiliations:** 1 Fukuoka Institute of Health and Environmental Sciences, Former affiliation: Department of Clinical Epidemiology, Institute of Industrial and Ecological Sciences, University of Occupational and Environmental Health, Japan.; 2 Department of Epidemiology and Environmental Health, Juntendo University School of Medicine.; 3 Department of Public Health, Fujita Health University School of Health Sciences.; 4 Department of Hygiene, Fujita Health University School of Medicine.; 5 Department of Preventive Medicine/Biostatistics and Medical Decision Making, Nagoya University Graduate School of Medicine.; 6 Department of Epidemiology for Community Health and Medicine, Kyoto Prefectural University of Medicine Graduate School of Medical Science.

Cancer prevention has been one of the major issues on health in Japan because 30% of total deaths are accounted for cancer.^1^ In order to clarify cancer risk, the Japan Collaborative Cohort Study (JACC Study) for Evaluation of Cancer Risk sponsored by the Ministry of Education, Science, Sports and Culture of Japan (Monbusho) was established in 1986 under the leadership of Dr. Kunio Aoki, Professor Emeritus, Nagoya University School of Medicine, and Dr. Haruo Sugano, the former Director of the Cancer Institute, Tokyo, with 36 active epidemiologists throughout Japan.^2^^-^^5^

Approximately 127,500 healthy inhabitants from 45 areas throughout Japan were enrolled into the study. Among this group, a total of 110,792 individuals aged 40-79 years, were followed up for a period of at least 10 years. Details of this cohort study was described elsewhere.^2^^-^^4^

Also, descriptive data for the JACC Study and validity studies were shown in the Journal of Epidemiology Supplement 1 in 2005,^5^ besides original papers published from the JACC Study.

In 1996, 10 years after the establishment of this cohort study, Professor Yoshiyuki Ohno, Nagoya University School of Medicine, succeeded Professor Kunio Aoki as chairman of the JACC study group. Under the leadership of Prof. Ohno, the JACC study was greatly supported by Dr. Tomoyuki Kitagawa, Chairman of Grant-in-Aid for Scientific Research on Priority Area ‘Cancer’, for serum analysis in 1998-99. Since 2000, granting system of the study has been changed. Now this study has been supported by the Committee of Scientific Research on Priority Areas of Cancer under the Chairman of the Committee, Dr. Kazuo Tajima, and conducted under the leadership of Dr. Akiko Tamakoshi. The granting system of the study is going to be changed again in 2005. Taking this opportunity, in the present supplement, authors intend to review present status of cancer sites (lung, stomach, pancreas and gall bladder, liver, colon and rectum, urinary tract, and esophagus) in Japan based on the data obtained from the JACC Study.

We hope that this supplement 2 of 2005 also help you to obtain latest descriptive data for each specific cancer site with the supplement 1 of the Journal of Epidemiology.

## MEMBER LIST OF THE JACC STUDY GROUP

The present investigators involved, with the co-authorship of this paper, in the JACC Study and their affiliations are as follows: Dr. Akiko Tamakoshi (present chairman of the study group), Nagoya University Graduate School of Medicine; Dr. Mitsuru Mori, Sapporo Medical University School of Medicine; Dr. Yutaka Motohashi, Akita University School of Medicine; Dr. Ichiro Tsuji, Tohoku University Graduate School of Medicine; Dr. Yosikazu Nakamura, Jichi Medical School; Dr. Hiroyasu Iso, Institute of Community Medicine, University of Tsukuba; Dr. Haruo Mikami, Chiba Cancer Center; Dr. Yutaka Inaba, Juntendo University School of Medicine; Dr. Yoshiharu Hoshiyama, University of Human Arts and Sciences; Dr. Hiroshi Suzuki, Niigata University School of Medicine; Dr. Hiroyuki Shimizu, Gifu University School of Medicine; Dr. Hideaki Toyoshima, Nagoya University Graduate School of Medicine; Dr. Kenji Wakai, Aichi Cancer Center Research Institute; Dr. Shinkan Tokudome, Nagoya City University Graduate School of Medical Sciences; Dr. Yoshinori Ito, Fujita Health University School of Health Sciences; Dr. Shuji Hashimoto, Fujita Health University School of Medicine; Dr. Shogo Kikuchi, Aichi Medical University School of Medicine; Dr. Akio Koizumi, Graduate School of Medicine and Faculty of Medicine, Kyoto University; Dr. Takashi Kawamura, Kyoto University Center for Student Health; Dr. Yoshiyuki Watanabe, Kyoto Prefectural University of Medicine Graduate School of Medical Science; Dr. Tsuneharu Miki, Graduate School of Medical Science, Kyoto Prefectural University of Medicine; Dr. Chigusa Date, Faculty of Human Environmental Sciences, Mukogawa Women’s University ; Dr. Kiyomi Sakata, Wakayama Medical University; Dr. Takayuki Nose, Tottori University Faculty of Medicine; Dr. Norihiko Hayakawa, Research Institute for Radiation Biology and Medicine, Hiroshima University; Dr. Takesumi Yoshimura, Fukuoka Institute of Health and Environmental Sciences; Dr. Akira Shibata, Kurume University School of Medicine; Dr. Naoyuki Okamoto, Kanagawa Cancer Center; Dr. Hideo Shio, Moriyama Municipal Hospital; Dr. Yoshiyuki Ohno, Asahi Rosai Hospital; Dr. Tomoyuki Kitagawa, Cancer Institute of the Japanese Foundation for Cancer Research; Dr. Toshio Kuroki, Gifu University; and Dr. Kazuo Tajima, Aichi Cancer Center Research Institute.
